# The WD40-Protein *Pf*WLP1 Ensures Stability of the *Pf*CCp-Based Adhesion Protein Complex in *Plasmodium falciparum* Gametocytes

**DOI:** 10.3389/fcimb.2022.942364

**Published:** 2022-07-18

**Authors:** Lena Roling, Ansgar Flammersfeld, Gabriele Pradel, Sandra Bennink

**Affiliations:** Division of Cellular and Applied Infection Biology, Institute of Zoology, RWTH Aachen University, Aachen, Germany

**Keywords:** malaria, *Plasmodium falciparum*, gametocyte, WD40-repeat protein, adhesion protein complex, CCp proteins

## Abstract

Members of the WD40-repeat protein family can be found in all eukaryotic proteomes where they usually serve as interaction platforms for the assembly of large protein complexes and are therefore essential for the integrity of these complexes. In the malaria parasite *Plasmodium falciparum*, the WD40-repeat protein *Pf*WLP1 has been shown to interact with members of distinct adhesion protein complexes in the asexual blood stages and gametocyte stages. In this study, we demonstrate that the presence of *Pf*WLP1 is crucial for both the stability of these gametocyte-specific adhesion complexes as well as for gametocyte maturation and gametogenesis. Using reverse genetics, we generated a *Pf*WLP1-knockdown parasite line for functional characterization of the protein. Knockdown of *Pf*WLP1 resulted in a slight reduction of gametocyte numbers and significantly the impaired ability of the gametocytes to exflagellate. *Pf*WLP1-knockdown further led to reduced protein levels of the *Limulus* coagulation factor C-like (LCCL)-domain proteins *Pf*CCp1 and *Pf*CCp2, which are key components of the adhesion complexes. These findings suggest that the interaction of *Pf*WLP1 with members of the *Pf*CCp-based adhesion complex ensures complex stability and thereby contributes to gametocyte viability and exflagellation.

## Introduction

The malaria parasite *Plasmodium falciparum* is transmitted from human to human through the bloodmeal of the mosquito vector. While symptoms in the human host are caused by asexual blood stage parasites, which multiply in the human red blood cells (RBCs), transmission to the mosquito is mediated by intraerythrocytic sexual precursor cells, the gametocytes. During their maturation in the human RBCs, the gametocytes pass through five morphologically distinguishable stages. When the mature gametocytes are taken up by the mosquito during its bloodmeal, they undergo sexual reproduction in the mosquito midgut and continue their life-cycle in the vector (reviewed in Kuehn and Pradel, 2010; Bennink et al., 2016).

The sexual phase of the malaria parasite goes along with the coordinated expression of stage-specific adhesion proteins, which assemble to complexes at the parasite plasma membrane. The complexes include members of the *P. falciparum* LCCL domain-containing protein family (*Pf*CCp) (Pradel et al., 2004; Pradel et al., 2006; Scholz et al., 2008; Simon et al., 2009; Simon et al., 2016; reviewed in Kuehn et al., 2010). The six *Pf*CCp proteins, which are highly conserved among the apicomplexan clade (Pradel et al., 2004; Becker et al., 2010; Bastos et al., 2013; reviewed in Dessens et al., 2004), interact by adhesion domain-mediated binding and assemble to a complex with adhesive properties. The *Pf*CCp-based protein complex is localized in the lumen of the parasitophorous vacuole (Pradel et al., 2004) and is anchored to the gametocyte plasma membrane *via* interactions with *Pf*s230, which itself is binding to the GPI-anchored protein *Pf*s48/45 (Kumar, 1987; Kumar and Wizel, 1992).

Upon gametocyte activation, coupling of the complex to the membrane is enhanced through the proteolytic processing of *Pf*s230, additional protein-protein-interactions and the integration of the adhesion protein *Pf*s25 into the complex, which further connects the complex with the plasma membrane *via* a GPI-anchor (Simon et al., 2016). These adhesion complexes are particularly present on the surface of the extracellular macrogametes that have formed during gametogenesis. It has been hypothesized that the *Pf*CCp-based adhesion complex might play a role in adhesive processes during fertilization such as mediating contact between the female macrogamete and male microgamete (reviewed in Kuehn et al., 2010).

The six *Pf*CCp proteins exhibit a co-dependent expression so that the lack of one *Pf*CCp protein leads to the loss of other members of the family, while *Pf*s230 expression is not affected (Simon et al., 2009). Further, the properly processed form of *Pf*s230 is necessary for the correct assembly of the complex, since incorrect processing or lack of *Pf*s230 leads to proteolysis of the *Pf*CCp proteins and release from the complex in activated gametocytes (Brooks and Williamson, 2000; Simon et al., 2016).

Previously, the WD40-repeat protein-like protein *Pf*WLP1 has been identified as a component of the *Pf*CCp-based protein complex in *P. falciparum* gametocytes (von Bohl et al., 2015). WD40-repeat protein-like proteins are characterized by WD40 domains, which in general are composed of seven or multiple of seven WD40-repeats. The repeats are short repeating amino acid motifs with a length of approximately 44 to 60 amino acids which are terminated by a tryptophan-aspartate (WD) dipeptide (reviewed in e.g. Voorn and Ploegh, 1992; Neer et al., 1994; Xu and Min, 2011). WD40-domain containing proteins act as scaffolds for the formation of large protein complexes and are involved in numerous biological processes such as signal transduction, cell division or transcription regulation depending on their interaction partners (reviewed in Stirnimann et al., 2010; Jain and Pandey, 2018).

In the *P. falciparum* genome, 80 putative WD40-domain containing proteins have been identified in an *in silico* analysis, most of which remain uncharacterized until now (Chahar et al., 2015). *Pf*WLP1, which has been demonstrated to be unique for the genus *Plasmodium*, has been shown to be expressed in the asexual schizont stage and during gametocyte development (von Bohl et al., 2015). In schizonts, the protein initially accumulates underneath the plasma membrane and subsequently relocalizes underneath the micronemes, when the merozoites have formed. In merozoites, *Pf*WLP1 then interacts with the transmembrane protein *Pf*AMA1 (apical membrane antigen 1) – a part of the AMA1/RON (rhoptry neck protein)-complex, which is involved in tight junction formation during RBC invasion (Lamarque et al., 2011; reviewed in Boucher and Bosch, 2015). In the sexual stages, *Pf*WLP1 primarily localizes underneath the plasma membrane and associates with *Pf*CCp1 and *Pf*s230 in maturing and activated gametocytes (von Bohl et al., 2015).

In this study we aimed to analyse in more detail the link between *Pf*WLP1 and the *Pf*CCp-based adhesion complex and to unveil the role of *Pf*WLP1 for gametocytes. To address the question, we generated a *glmS*-based *Pf*WLP1-knockdown (KD) line and functionally characterized the protein using biochemical and cell-based assays.

## Methods

### Gene Identifiers

The following PlasmoDB gene identifiers are assigned to the proteins investigated in this study: *Pf*39 [PF3D7_1108600], *Pf*CCp1 [PF3D7_1475500], *Pf*CCp2 [PF3D7_1455800], *Pf*s230 [PF3D7_0209000], *Pf*WLP1 [PF3D7_1443400].

### Antibodies

The following antisera were used in this study: mouse polyclonal antisera against *Pf*CCp1rp1 (Scholz et al., 2008), *Pf*s230 region C (Williamson et al., 1995), *Pf*39rp1 (Simon et al., 2009) and *Pf*WLP1rp1 (von Bohl et al., 2015); rabbit polyclonal antisera against *Pf*CCp2 (Simon et al., 2009) and *Pf*s230 (Biogenes); and polyclonal rabbit anti-HA antibody (Sigma-Aldrich).

### Parasite Lines

In this study, the *Plasmodium falciparum* wild-type (WT) strain NF54 (ATCC) was used. The generation of the *Pf*WLP1-KD line is described below.

### Parasite Culture

The asexual blood stage parasites and gametocytes of the WT strain NF54 (WT NF54) and the *Pf*WLP1-KD line were cultivated in human A+ RBCs in RPMI1640/HEPES medium (Gibco) supplemented with 10% v/v heat-inactivated human serum (A+). The medium was further complemented with 50 µg/ml hypoxanthine (Sigma-Aldrich) and 10 µg/ml gentamicin (Gibco). For cultivation of the *Pf*WLP1-KD line, the selection drug WR99210 (Jacobus Pharmaceutical Company) was added in a final concentration of 2.5 nM. The cultures were kept at 37°C in an atmosphere of 5% O_2_ and 5% CO_2_ in N_2_. Human A+ RBCs and sera were purchased from the Institute of Transfusion Medicine, University Hospital Aachen, Germany. The donors remained anonymous and the RBC and serum samples were pooled. The work on human blood was approved by the Ethics Commission of RWTH Aachen University. Synchronization of ring stage parasites was performed using sorbitol treatment as described (Lambros and Vanderberg, 1979). Gametocyte stages were enriched by Percoll gradient centrifugation (GE Healthcare Life Science) as previously described (Kariuki et al., 1998). *In vitro* induction of gametogenesis was performed by incubating mature gametocytes in 100 µM xanthurenic acid dissolved in 1% v/v 0.5 M NH_4_OH/ddH_2_O for 15 min at RT.

### Generation of *Pf*WLP1-KD and Induction of Knockdown

The *Pf*WLP1-KD line was generated by single-crossover homologous recombination using the pARL-HA-*glmS* vector (Flammersfeld et al., 2020). A 1053-bp fragment homologous to the 3’ end of the *pfwlp1* gene was amplified *via* PCR using forward primer 5’-TAGCGCGGCCGCGAATTCTAAAAATATGGCTACCTAC-3’ and reverse primer 5’-TACGCCCTAGGAAAAGCCACAAACGCCCAGAG-3’. Ligation of insert and vector backbone was mediated by NotI and AvrII restriction sites that were added to the PCR product *via* the primers. A synchronized WT NF54 ring stage culture was electroporated with 100 µg of the plasmid in transfection buffer (310 V, 950 µF, 12 ms; Bio-Rad gene pulser Xcell) as described (Wirth et al., 2014). WR99210 was added to the culture in a final concentration of 2.5 nM starting 6 h after transfection. Once resistant parasites appeared in the culture, they were checked for plasmid integration into the genome by diagnostic PCR. Therefore, genomic DNA of the transfected cultures was isolated using the NucleoSpin Blood Kit (Macherey-Nagel) and used as a template in the PCR. The following primers were used in the PCR (see **Figure S1A** for regions of primer binding): *Pf*WLP1-KD-5’ integration forward primer 5’-CAATATATTAATGACAAGCGGTTATGATGG-3’ (primer 1), *Pf*WLP1-KD-3’ integration reverse primer 5’-GTATATAATTTTCATGTTTTTAATATTGTACTCTC-3’ (primer 2), pARL-HA-*glmS* forward primer 5’-GCTTTACACTTTATGCTTCCGGCTCG-3’ (primer 3) and pARL-HA-*glmS* reverse primer 5’-CCTTAGAGCTCGGCATAATCTGG-3’ (primer 4). To induce the *glmS*-ribozyme and thereby the knockdown of *pfwlp1* gene expression, the culture was treated with 2.5 mM glucosamine hydrochloride (GlcN; D-(+)-glucosamine hydrochloride; Sigma-Aldrich) as described (Prommana et al., 2013).

### Growth Assays and Exflagellation Assay

To investigate the asexual blood stage replication and gametocyte development, cultures of the WT NF54 strain or the *Pf*WLP1-KD line were synchronized and set to an initial parasitemia of 0.25% or 2%, respectively. The parasites were cultivated either in the presence or absence of 2.5 mM GlcN. For the asexual growth assay, samples were taken and Giemsa-stained thin blood smears were prepared every 12 h over a time period of 96 h. For the gametocyte development assay, smears were prepared at five or six different time points over a time period of 13 to 15 d. The smears were evaluated microscopically at 1,000-fold magnification and parasitemia and gametocytemia were calculated by counting the percentage of parasites or gametocytes in 1,000 RBCs in triplicate, respectively. For the comparative exflagellation assay, gametocytes of the WT NF54 strain and the *Pf*WLP1-KD line were cultivated in presence or absence of 2.5 mM GlcN for 12 to 14 days. Two days before the execution of the assay, GlcN was withdrawn from the cultures, since no exflagellation can be observed in GlcN-containing medium. The gametocytemia was adjusted between the samples to be compared and 100 µl of each culture was activated *in vitro* as described above. At 15 min post-activation, numbers of exflagellation centers were counted microscopically at 400-fold magnification in 30 optical fields.

### Western Blot Analysis

While asexual blood stage parasites from WT NF54 and the *Pf*WLP1-KD line were harvested from either mixed or synchronized cultures, gametocytes were enriched *via* Percoll gradient centrifugation (see above). Parasites were released from the enveloping RBC membrane by incubation in 0.05% w/v saponin/PBS for 10 min. Parasites were pelleted and resuspended in lysis buffer (150 mM NaCl, 0.1% v/v Triton X-100, 0.5% w/v sodium deoxycholate, 0.1% w/v SDS, 50 mM Tris-HCl pH 8.0) supplemented with protease inhibitor cocktail (complete EDTA-free, Roche). The lysates were supplemented with 5x SDS-PAGE loading buffer containing 25 mM dithiothreitol and heat-denatured at 95°C for 10 min. The protein lysates were separated *via* SDS-PAGE and subsequently transferred to a Hybond ECL nitrocellulose membrane (Amersham Biosciences). Blocking of non-specific binding sites was performed by incubation of the membrane in Tris-buffered saline containing 5% w/v skim milk, pH 7.5. For immunodetection, the membrane was incubated with polyclonal mouse anti-*Pf*39 antisera (dilution 1:2,500), mouse anti-*Pf*CCp1 antisera (dilution 1:1,000), rabbit anti-*Pf*CCp2 antisera (dilution 1:1,000) or rabbit anti-HA antibody (1:5,000) diluted in blocking solution at 4°C overnight. The membrane was washed and further incubated for 1 h at RT with a goat anti-mouse or anti-rabbit alkaline phosphatase-coupled secondary antibody (dilution 1:10,000, Sigma-Aldrich) and developed in a solution of nitroblue tetrazolium chloride (NBT) and 5-bromo-4-chloro-3-indoxylphosphate (BCIP, Sigma-Aldrich) for 5-15 min at RT. After being scanned, Western blots (WBs) were processed and band intensities were measured using the ImageJ 1.51f software.

### Indirect Immunofluorescence Assay

Untreated and GlcN-treated gametocytes of the *Pf*WLP1-KD line were air-dried as cell monolayers on glass slides and fixed in methanol at -80°C for 10 min. Membrane permeabilization and blocking of non-specific binding sites was performed by incubation in 0.01% w/v saponin/0.5% w/v BSA/PBS with 1% v/v neutral goat serum for 30 min at RT. For immunostaining, the preparations were incubated for 2 h at 37°C with polyclonal mouse anti-*Pf*WLP1 antiserum (dilution 1:50), polyclonal mouse anti-*Pf*CCp1 antiserum (dilution 1:50) or polyclonal rabbit anti-*Pf*CCp2 antiserum (dilution 1:200) diluted in blocking solution. After washing, binding of the primary antibody was detected by incubation with Alexa Flour 488-conjugated goat anti-mouse or goat anti-rabbit secondary antibody (Invitrogen) diluted 1:1,000 in PBS for 45 min at 37°C. Counterstaining of the gametocyte plasma membrane was performed by incubation with polyclonal rabbit anti-*Pf*s230 antiserum (dilution 1:500) or polyclonal mouse anti-*Pf*s230 antiserum (dilution 1:300) in blocking solution for 1 h at 37°C, followed by incubation for 45 min at 37°C with polyclonal Alexa Flour 594-conjugated goat anti-rabbit or goat anti-mouse secondary antibody (Invitrogen) diluted 1:1,000 in PBS. Parasite nuclei were highlighted by treatment with Hoechst 33342 nuclear stain (Invitrogen) for 1 min at RT. Cells were mounted with anti-fading solution AF2 (CitiFlour™) and sealed with nail varnish. Microscopic evaluation was performed using a Leica DM 5500 B fluorescence microscope and fluorescence intensity was quantified using ImageJ 1.51f. Images were processed using Adobe Photoshop CS software.

## Results

### Generation of a *Pf*WLP1-KD Parasite Line

**Figure 1:**
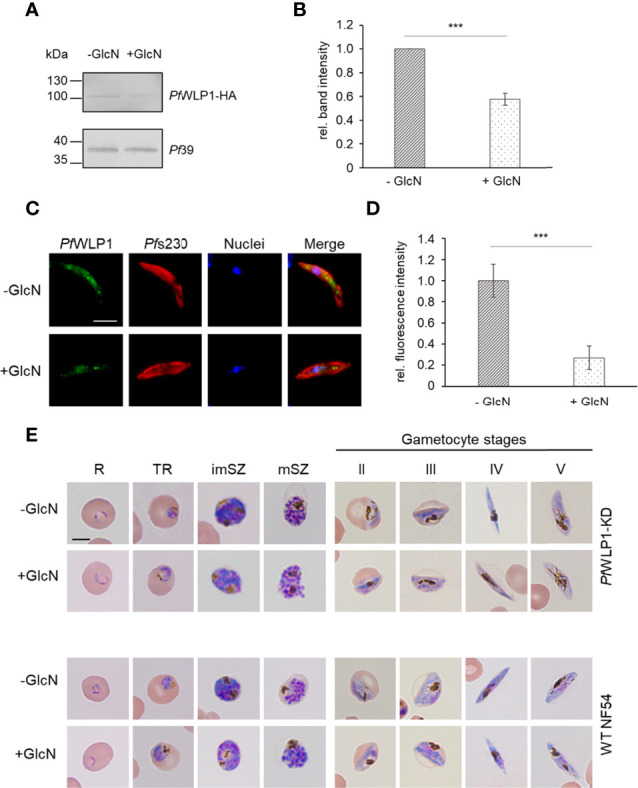
Knockdown of *Pf*WLP1 does not affect parasite morphology. **(A)** Downregulation of *Pf*WLP1-HA expression in *Pf*WLP1-KD gametocytes. Gametocytes of the *Pf*WLP1-KD line were cultivated either in the presence or absence of 2.5 mM GlcN for 14 d. Protein lysates were subjected to WB using rabbit anti-HA antibody to detect *Pf*WLP1-HA (~108 kDa). Equal loading was confirmed using a polyclonal mouse anti-*Pf*39 antiserum (~39 kDa). **(B)** Quantification of the *Pf*WLP1-HA expression in *Pf*WLP1-KD gametocytes. The intensities of the *Pf*WLP1-HA specific protein bands of three independent WB (performed as described in A) were quantified using ImageJ 1.51f and normalized to the respective band intensities for *Pf*39 (mean ± SD; untreated set to 1). ***p < 0.001 (Student’s t-test). **(C)** Immunolabeling of *Pf*WLP1-HA in gametocytes of the *Pf*WLP1-KD line. Gametocytes of the *Pf*WLP1-KD line were cultivated as described in **(A)** Untreated and GlcN-treated gametocytes were immunolabeled with polyclonal mouse anti-*Pf*WLP1 antiserum (green) and counterstained with polyclonal rabbit anti-*Pf*s230 antiserum (red). Nuclei were highlighted with Hoechst 33342 nuclear stain (blue). Bar, 5 µm. **(D)** Quantification of the *Pf*WLP1-HA fluorescence signal in *Pf*WLP1-KD gametocytes in the presence or absence of GlcN. IFAs were performed as described in **(C)** The fluorescence intensity of the *Pf*WLP1-HA signal was quantified in 20 gametocytes using ImageJ 1.51f (mean ± SD; untreated set to 1). ***p < 0.001 (Student’s t-test). **(E)** Morphology of *Pf*WLP1-KD blood stage parasites. Asexual blood stage parasites and gametocytes of WT NF54 and *Pf*WLP1-KD were cultivated either in the presence or absence of 2.5 mM GlcN. Cultures were treated with GlcN for 3 d (asexual blood stages) or 5-14 d (gametocyte stages). The morphology was analyzed *via* Giemsa staining of blood smears. R, ring; TR, trophozoite; imSZ, immature schizont; mSZ, mature schizont. Bar, 5 µm. The results **(A, C, E)** are representative for three independent experiments.

*Pf*WLP1 was previously shown to be a 96-kDa cytosolic protein comprising five WD40 motifs that accumulates underneath the plasma membrane in gametocytes and here associates with members of the *Pf*CCp-based adhesion protein complex (von Bohl et al., 2015). So far, the generation of a *Pf*WLP1-knockout (KO) parasite line remains unsuccessful, although genetic manipulation of the *pfwlp1* gene locus is possible (von Bohl et al., 2015). To characterize the role of *Pf*WLP1 in the formation of the gametocyte specific adhesion protein complex in more detail, an inducible *Pf*WLP1-KD line was generated, using the previously described pARL-HA-*glmS* vector (Flammersfeld et al., 2020). The pARL-HA-*glmS*-WLP1 vector was electroporated into ring stage parasites and WR99210 resistant parasites were obtained after six weeks. Integration of the vector into the *pfwlp1* gene locus was confirmed by integration PCR (**Figures S1A, B**). Successful downregulation of *pfwlp1* gene expression and in consequence reduced protein levels were confirmed by WB. For this, mixed asexual blood parasites of the *Pf*WLP1-KD line were cultivated in the presence or absence of 2.5 mM GlcN for three days. Subsequent WB using a polyclonal anti-HA antibody confirmed the presence of the *Pf*WLP1-HA fusion protein in lysates of the mutant line. Further, treatment with GlcN led to a reduction of *Pf*WLP1-HA protein levels by approximately 45% (**Figures S1C, D**). Immunoblotting with polyclonal mouse anti-*Pf*39 antisera was used as a loading control and for normalization of band intensities. To confirm the downregulation of *Pf*WLP1 in the gametocyte stages, gametocytes of the *Pf*WLP1KD-line were cultivated in the presence or absence of 2.5 mM GlcN for 14 d, followed by WB analysis and quantification of the *Pf*WLP1-HA-specific band intensities was performed as described above. Similar to the asexual blood stages, GlcN-treatment in gametocytes led to a significant reduction of *Pf*WLP1-HA protein levels by approximately 40% (**Figures 1A, B**). Reduction of *Pf*WLP1-HA protein levels in gametocytes upon GlcN-treatment was further confirmed *via* immunofluorescence assay (IFA), where even a reduction of the protein levels by approximately 75% was detected (**Figures 1C, D**). To investigate, if the reduction of the *Pf*WLP1-HA protein levels affect the morphology of the parasites, thin blood smears were prepared from WT NF54 and *Pf*WLP1-KD cultures that were cultivated either in the presence or absence of 2.5 mM GlcN. The smears were Giemsa-stained and evaluated by light microscopy. The treatment with GlcN did not have a visible effect on the morphology of either WT NF54 or *Pf*WLP1-KD blood stage parasites. Both, asexual blood stages and gametocytes at different stages of maturity exhibited normal morphologies after GlcN-treatment (**Figure 1E**).

### *Pf*WLP1 Is Crucial for Exflagellation

**Figure 2:**
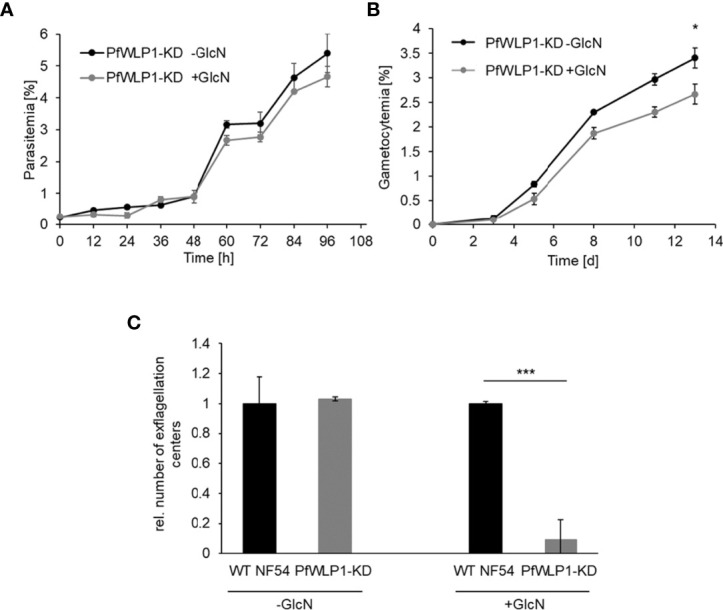
Parasite development and exflagellation in the *Pf*WLP1-KD line. **(A)** Asexual blood stage replication of *Pf*WLP1-KD parasites. Synchronized ring stage parasites of the *Pf*WLP1-KD line with a starting parasitemia of 0.25% were cultivated either in the presence or absence of 2.5 mM GlcN for 96 h. The parasitemia was evaluated microscopically every 12 h using Giemsa smears. The experiment was performed in triplicate (mean ± SD). **(B)** Gametocyte formation of *Pf*WLP1-KD parasites. Synchronized ring stage parasites of the *Pf*WLP1-KD line with a starting parasitemia of 2% were cultivated either in the presence or absence of 2.5 mM GlcN over a time period of 13 d. The numbers of gametocytes were evaluated microscopically on 5 time points using Giemsa smears. The experiment was performed in triplicate (mean ± SD). The differences in gametocytemia between the treated and untreated culture on day 13 were investigated statistically (*p < 0.05; Student’s t-test). **(C)** Formation of exflagellation centers in the *Pf*WLP1-KD line. WT NF54 and *Pf*WLP1-KD gametocytes were cultivated in 2.5 mM GlcN. Two days before the exflagellation assay, GlcN was withdrawn from the culture. The gametocytes were induced *in vitro* and 15 min after activation the numbers of exflagellation centers were evaluated microscopically in 30 optical fields. Untreated cultures served as control. The experiment was performed in triplicate (mean ± SD; numbers of exflagellation centers in WT NF54 cultures were set to 1). ***p < 0.001 (Student’s t-test). The results **(A–C)** are representative for three independent experiments.

Comparative phenotype analyses between WT NF54 and the *Pf*WLP1-KD line cultivated either in the presence or absence of 2.5 mM GlcN showed that downregulation of *Pf*WLP1-HA does not have a significant effect on the asexual blood stage replication (**Figures 2A**, **Figure S1E**). However, GlcN-treated *Pf*WLP1-KD parasites showed a significant reduction in gametocyte numbers formed after 13 d of cultivation compared to untreated parasites (**Figure 2B**), while GlcN-treatment did not affect gametocyte formation in WT NF54 parasites (**Figure S1F**), indicating that *Pf*WLP1 impacts gametocyte maturation. Hence, the ability of the parasites to form motile male microgametes was analyzed in exflagellation assays. Therefore, WT NF54 and *Pf*WLP1-KD gametocytes were grown in GlcN-containing medium until maturity. Two days prior to gametocyte activation, GlcN was withdrawn from the cultures. After *in vitro* activation of the gametocytes, the numbers of exflagellation centers were evaluated by light microscopy. GlcN-treatment resulted in a significant inhibition of exflagellation in *Pf*WLP1-KD gametocytes when compared to GlcN-treated WT NF54 gametocytes (**Figure 2C**), pointing to a crucial role of *Pf*WLP1 in exflagellation. Untreated *Pf*WLP1-KD and WT NF54 gametocytes on the other hand exhibited comparable numbers of exflagellation centers in this experiment (**Figure 2C**).

### Knockdown of *Pf*WLP1 Leads to Reduced Expression of Members of the *Pf*CCp-Complex

**Figure 3:**
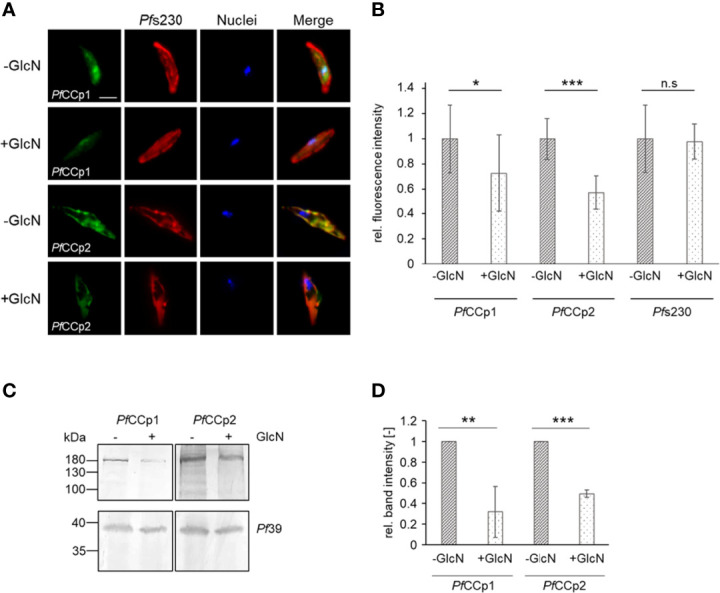
*Pf*WLP1-KD affects protein abundance of *Pf*CCp1 and *Pf*CCp2. **(A)** Immunolabeling of *Pf*CCp1 and *Pf*CCp2 in gametocytes of the *Pf*WLP1-KD line. Gametocytes of the *Pf*WLP1-KD line were cultivated either in the presence or absence of GlcN for 14 d. Untreated and GlcN-treated gametocytes were immunolabeled with polyclonal mouse anti-*Pf*CCp1 antiserum or polyclonal rabbit anti-*Pf*CCp2 antiserum (green) and counterstained with polyclonal rabbit or mouse anti-*Pf*s230 antisera (red). Nuclei were highlighted with Hoechst 33342 nuclear stain (blue). Bar, 5 µm. **(B)** Immunolabeling has been performed as described in **(A)** The fluorescence intensity of *Pf*CCp1, *Pf*CCp2 and *Pf*s230 signals was quantified in 20 gametocytes using ImageJ 1.51f (mean ± SD; untreated set to 1). *p < 0.05; ***p < 0.001; n.s., not significant (Student’s t-test). The results are representative for two independent experiments. **(C)** Gametocytes of the *Pf*WLP1-KD line were cultivated as described in **(A)** Gametocyte protein lysates were subjected to WB and *Pf*CCp1 (~185 kDa) and *Pf*CCp2 (~185 kDa) were immunolabeled with polyclonal mouse anti-*Pf*CCp1 and rabbit anti-*Pf*CCp2 antisera, respectively. Equal loading was confirmed using a polyclonal mouse anti-*Pf*39 antiserum (~39 kDa). **(D)** Quantification of the *Pf*CCp1 and *Pf*CCp2 expression in *Pf*WLP1-KD gametocytes. The intensities of the *Pf*CCp1 and *Pf*CCp2 specific protein bands of three independent WB (performed as described in C) were quantified using ImageJ 1.51f and normalized to the respective band intensities for *Pf*39 (mean ± SD; untreated set to 1). **p < 0.01; ***p < 0.001 (Student’s t-test). The results **(A, C, D)** are representative for three independent experiments.

Previous studies unveiled a co-localization and interaction of *Pf*WLP1 with members of the *Pf*CCp-complex (von Bohl et al., 2015). With the help of the *Pf*WLP1-KD line we now aimed at investigating a potential effect of *Pf*WLP1 on the integrity and stability of the *Pf*CCp-based adhesion protein complex. Therefore, gametocytes of the *Pf*WLP1-KD line were treated with GlcN to induce KD of *Pf*WLP1 and protein levels of selected members of the *Pf*CCp-complex (i.e. *Pf*CCp1 and *Pf*CCp2) were evaluated. In IFA, immunolabelling for both, *Pf*CCp1 and *Pf*CCp2 was significantly reduced in GlcN-treated *Pf*WLP1-KD gametocytes compared to untreated gametocytes (**Figures 3A, B**). However, the expression pattern analysis showed a typical punctuated localization of the remaining *Pf*CCp1 and *Pf*CCp2 on the gametocyte plasma membrane, as it was previously described (Pradel et al., 2004; Pradel et al., 2006). Noteworthy, the immunolabeling for *Pf*s230, which was used for counterstaining, did not alter between the treated and untreated gametocytes, although it is also part of the *Pf*CCp-protein complex (Scholz et al., 2008; Simon et al., 2009; reviewed in Kuehn et al., 2010). In WT NF54 gametocytes, GlcN-treatment did not have an effect on the intensity of *Pf*CCp1- and *Pf*CCp2-fluorescence signals (**Figures S2A, B**). *Pf*CCp1 and *Pf*CCp2 protein levels were further compared in protein lysates of GlcN-treated and untreated *Pf*WLP1-KD gametocytes *via* WB followed by quantification of band intensities. Immunoblotting and quantification revealed that the *Pf*CCp1- and *Pf*CCp2-specific protein bands were significantly less intense in the GlcN-treated samples compared to the untreated samples (**Figures 3C, D**). Immunoblotting with antisera against *Pf*39 served as a loading control and was used for normalization of the band intensities. The combined data demonstrate that the lack of *Pf*WLP1 results in a reduction of *Pf*CCp proteins on the gametocyte plasma membrane.

## Discussion

Apicomplexan parasites exhibit various types of adhesive protein complexes on the cell membranes, which exert distinct functions and which depend on the life-cycle stage of the parasite. These adhesive multi-protein complexes are utilized by the parasites for different types of intercellular contact, ranging from gliding and attachment to target cells to host cell invasion and cytoadherence of infected RBCs. In the malaria parasite *P. falciparum*, membrane-coupled protein complexes composed of transmembrane proteins as well as GPI-anchored and peripheral adhesion proteins are known for the different life-cycle stages of the parasite. One example is the merozoite surface protein (MSP)-based protein complex, which is essential for the successful attachment of the merozoites to RBCs prior to invasion (reviewed in Cowman and Crabb, 2006). Merozoites further express the *Pf*AMA1/RON complex, which defines the merozoite-RBC moving junction (e.g. Collins et al., 2009; Richard et al., 2010; Srinivasan et al., 2011). *Pf*AMA1 is stored in the micronemes and translocated to the merozoite surface before invasion, while at the same time, the RON-complex is inserted into the host cell membrane (Cao et al., 2009; Riglar et al., 2011; reviewed in Shen and Sibley, 2012).

An adhesion complex similar in its structure to the *Pf*MSP-based complex is found in gametocytes and gametes. The multi-protein complex is based on the six members of the LCCL-domain/*Pf*CCp protein family and further includes the 6-cys proteins *Pf*s230 and *Pf*s48/45, the latter of which is linked to the membrane *via* a GPI anchor. In addition, the EGF-domain protein *Pf*s25 links the complex to the macrogamete surface (e.g. Simon et al., 2009; Simon et al., 2016; reviewed in Kuehn et al., 2010). The adhesive *Pf*CCp-based protein complex on the surface of the gametes is crucial for sexual reproduction. Interestingly, (KO) of the *Pf*CCp-proteins does not abolish exflagellation in the respective KO-lines, but impairs the transition of midgut sporozoites to the salivary glands (Pradel et al., 2004; Scholz et al., 2008). *Pf*s230-KO parasites are also able to egress from the host RBC and exflagellate, but are unable to interact with surrounding cells and form exflagellation centers (Eksi et al., 2006).

In addition to the adhesion proteins on the gametocyte plasma membrane, the cytosolic WD40-domain protein *Pf*WLP1 has previously been described as an interaction partner of the *Pf*CCp-protein complex (von Bohl et al., 2015). *Pf*WLP1 is a member of the WD40-domain containing protein family which is one of the most abundant protein classes in eukaryotes and also includes prokaryotic proteins. Out of the 80 putative WD40-domain-containing proteins that have been identified in the *P. falciparum* genome (Chahar et al., 2015), only a few have been partially characterized so far, including for example the nuclear pore complex component *Pf*Sec13, which is also involved in the biogenesis of COPII-coated vesicles (Dahan-Pasternak et al., 2013), *Pf*RACK, an ortholog of the receptor for activated C kinase (Madeira et al., 2003) and the histone chaperone *Pf*RbAp46/48 (Kaushik et al., 2020). *Pf*WLP1 was shown to be expressed in the maturing schizont as well as the gametocyte stages, where it accumulates underneath the plasma membrane and interacts with the stage-specific *Pf*AMA1/RON- and *Pf*CCp-complexes, respectively (von Bohl et al., 2015).

In this study we have successfully generated a *Pf*WLP1-KD line, in which the *Pf*WLP1 protein levels can be reduced by approximately 40-45% *via* GlcN-mediated transcript degradation, as detected in WB. The KD did not show any growth defect during the asexual replication cycle, pointing to a non-essential role of *Pf*WLP1 in schizogony, contrary to assumptions in previous studies, in which a gene-KO remained unsuccessful (von Bohl et al., 2015) and a *piggyBac* transposon mutagenesis study that confirmed essentiality of *Pf*WLP1 (Zhang et al., 2018). These contradictory data might be explained by the low KD rate, in asexual blood stages after 3 d of GlcN-treatment. Longer GlcN-treatment could lead to higher KD rates which might result in growth defects.

In the *Pf*WLP1-KD, gametocyte maturation was affected and exflagellation was severely impaired when compared to WT NF54 parasites. Interestingly, the exflagellation ability is not affected in KOs of *Pf*CCp1 – an interaction partner of *Pf*WLP1 – or other *Pf*CCp proteins. Noteworthy, antibodies against *Pf*CCp1-4 and *Pf*FNPA are able to inhibit exflagellation, however this effect is only measurable in active serum and not in heat-inactivated serum, pointing to a participation of active complement in the process (Scholz et al., 2008).

We further showed that the KD of *Pf*WLP1 leads to reduced abundance of members of the *Pf*CCp-complex, i.e. its interaction partners *Pf*CCp1 and *Pf*CCp2. Noteworthy, in the GlcN-treated *Pf*WLP1-KD the remaining *Pf*CCp1 and *Pf*CCp2 proteins showed a similar subcellular localization as in the untreated culture, indicating that the *Pf*CCp-complex still assembles and localizes correctly in the *Pf*WLP1-KD line. However, low levels of *Pf*CCp proteins are still present on the gametocyte surface, which might also be explained by the fact, that only roughly 45% of *Pf*WLP1 was downregulated. Interestingly, the expression of *Pf*s230, which is also a member of the *Pf*CCp-based protein complex, was not affected in the *Pf*WLP1-KD. Similar results were obtained when the co-dependent expression of the *Pf*CCp-proteins was analysed. While KO of one *Pf*CCp-protein led to the complete or partial loss of other members of the protein family, the expression and localization of *Pf*s230 was not affected, indicating that *Pf*s230 expression is not dependent on the presence and assembly of all complex members (Pradel et al., 2006; Simon et al., 2009; reviewed in Kuehn et al., 2010).

**Figure 4:**
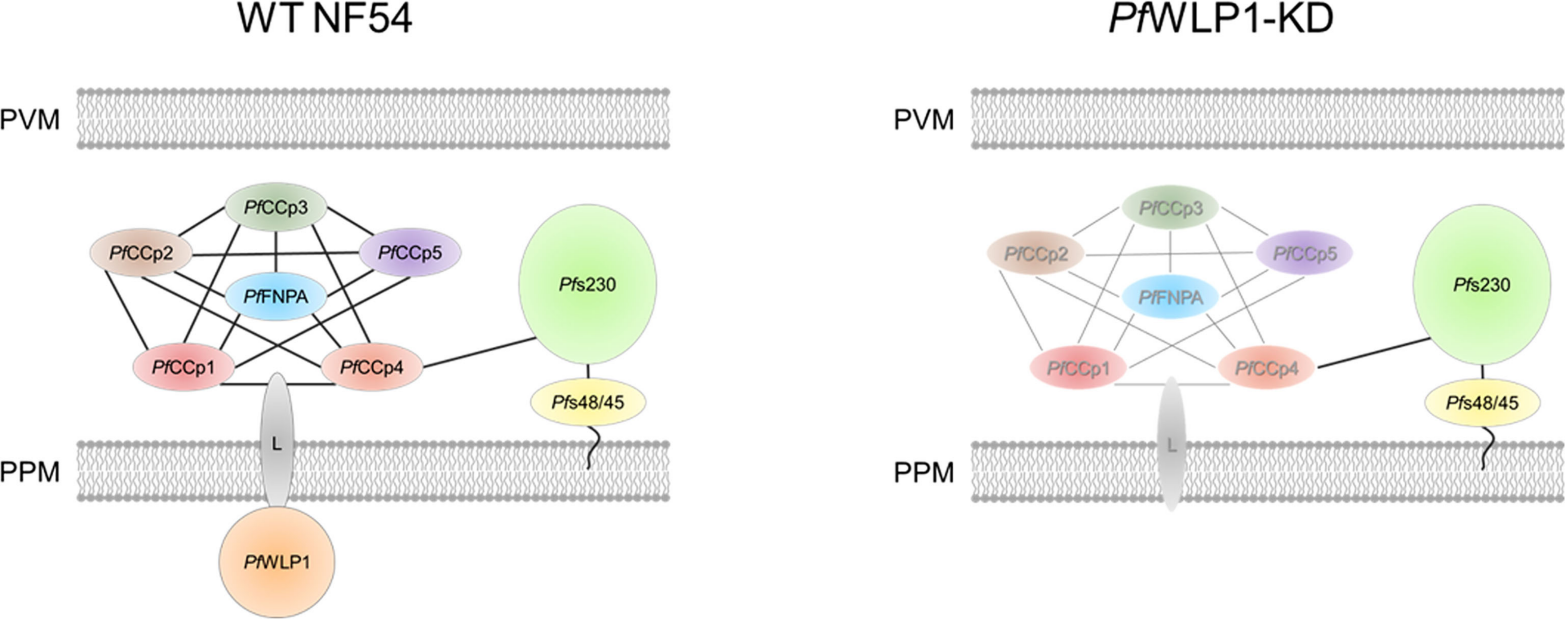
Model of the composition of the *Pf*CCp-based protein complex in WT NF54 gametocytes and proposed changes in *Pf*WLP1-KD gametocytes. Intracellular *Pf*WLP1 probably binds to the *Pf*CCp-complex *via* an unknown membrane-spanning linker protein (L). In the absence of *Pf*WLP1, the *Pf*CCp-complex is destabilized, while *Pf*s230 remains unaffected (destabilized complex shown in grey). PPM, parasite plasma membrane; PVM, parasitophorous vacuole membrane.

Based on these data we suggest that the cytosolic *Pf*WLP1 serves as an interaction platform for the *Pf*CCp-based adhesion protein complex in gametocytes and gametes and is important for its assembly and/or integrity. We hypothesize that the intracellular *Pf*WLP1 binds to the *Pf*CCp-complex *via* an unknown membrane-spanning linker protein, which binds to one or more *Pf*CCp-proteins in the lumen of the parasitophorous vacuole. The complex is bound to *Pf*s230 *via* interactions of *Pf*s230 and *Pf*CCp4 (see **Figure 4**). As *Pf*WLP1 was described to localize in close proximity with sub-pellicular proteins (von Bohl et al., 2015), it might connect the membrane-linked protein complex with the inner membrane complex of the parasite.

Since several members of the *Pf*CCp-complex have been proposed and investigated as putative antigens for the development of transmission-blocking vaccines, further analysis of the constituents and interactions of this complex is of particular importance.

## Data Availability Statement

The original contributions presented in the study are included in the article/**Supplementary Material**. Further inquiries can be directed to the corresponding author.

## Author Contributions

LR: methodology and data analysis. AF: methodology and data analysis. SB: data analysis and visualization. GP: conception and project administration. SB and GP: writing original draft. All authors contributed to the article and approved the submitted version.

## Funding

This work was supported by the Deutsche Forschungsgemeinschaft (PR905/15-1 to GP).

## Conflict of Interest

The authors declare that the research was conducted in the absence of any commercial or financial relationships that could be construed as a potential conflict of interest.

## Publisher’s Note

All claims expressed in this article are solely those of the authors and do not necessarily represent those of their affiliated organizations, or those of the publisher, the editors and the reviewers. Any product that may be evaluated in this article, or claim that may be made by its manufacturer, is not guaranteed or endorsed by the publisher.
