# Supplementary material for: The WD40-Protein PfWLP1 Ensures Stability of the PfCCp-Based Adhesion Protein Complex in Plasmodium falciparum Gametocytes
Source: Front Cell Infect Microbiol. 2022 Jul 18;12:942364. doi: 10.3389/fcimb.2022.942364 (PMC9339629; doi:10.3389/fcimb.2022.942364)
Supplement: Supplementary file 1 [file DataSheet_1.docx]

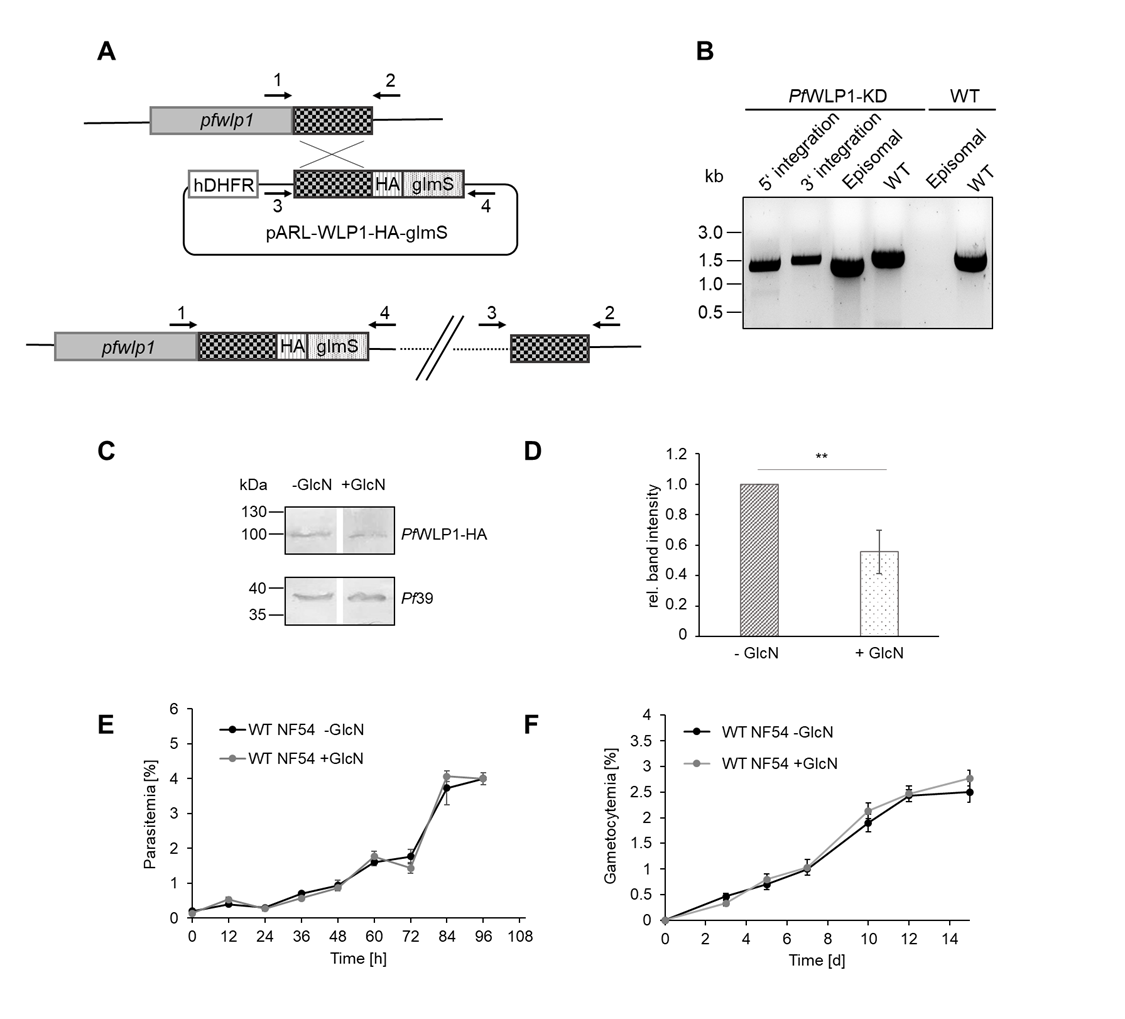


**Fig. S1** Generation of the *Pf*WLP1-KD line. **A.** Schematic depicting the single-crossover homologous integration strategy to generate the *Pf*WLP1-HA-*glmS* knockdown line. Numbered arrows indicate the position of primers used in the integration PCR. **B.** Verification of gene locus integration of the pARL-WLP1-HA-*glmS* vector. Primers 1 and 4 were used for amplification of the 5’-integration (1329 bp) and primers 3 and 2 for 3’-integration (1436 bp) of the vector. WT *pfwlp1* locus was amplified using primers 1 and 2 (1500 bp) and episomal DNA was amplified with primers 3 and 4 (1265 bp). Isolated gDNA from mock control (WT) was used for negative control. **C.** Downregulation of *Pf*WLP1-HA expression in asexual blood stages of the *Pf*WLP1-KD. Mixed asexual blood stage parasites of the *Pf*WLP1-KD line were cultivated either in the presence or absence of 2.5 mM GlcN for 3 d. Protein lysates were subjected to WB using rabbit anti-HA antibody to detect *Pf*WLP1-HA (~108 kDa). Equal loading was confirmed using a polyclonal mouse anti-*Pf*39 antiserum (~39 kDa). **D.** Quantification of the *Pf*WLP1-HA expression in asexual blood stage parasites of the *Pf*WLP1-KD line. The intensities of the *Pf*WLP1-HA specific protein bands of three independent WB (performed as described in C) were quantified using ImageJ 1.51f and normalized to the respective band intensities for *Pf*39 (mean ± SD; untreated set to 1). ** p < 0.01 (Student’s t-test). **E.** Asexual blood stage replication of WT NF54 parasites. Synchronized WT NF54 ring stage parasites with a starting parasitemia of 0.25 % were cultivated either in the presence or absence of 2.5 mM GlcN for 96 h. The parasitemia was evaluated microscopically every 12 h using Giemsa smears. The experiment was performed in triplicate (mean ± SD). **F.** Gametocyte formation of WT NF54 parasites. Synchronized WT NF54 ring stage parasites with a starting parasitemia of 2 % were cultivated either in the presence or absence of 2.5 mM GlcN over a time period of 15 d. The numbers of gametocytes were evaluated microscopically on 6 time points using Giemsa smears. The experiment was performed in triplicate (mean ± SD). The differences in gametocytemia between the treated and untreated culture on day 15 were statistically not significant (p > 0.05; Student’s t-test). The results (B-F) are representative for three independent experiments.
