# Supplementary material for: The WD40-Protein PfWLP1 Ensures Stability of the PfCCp-Based Adhesion Protein Complex in Plasmodium falciparum Gametocytes
Source: Front Cell Infect Microbiol. 2022 Jul 18;12:942364. doi: 10.3389/fcimb.2022.942364 (PMC9339629; doi:10.3389/fcimb.2022.942364)
Supplement: Supplementary file 2 [file DataSheet_2.docx]

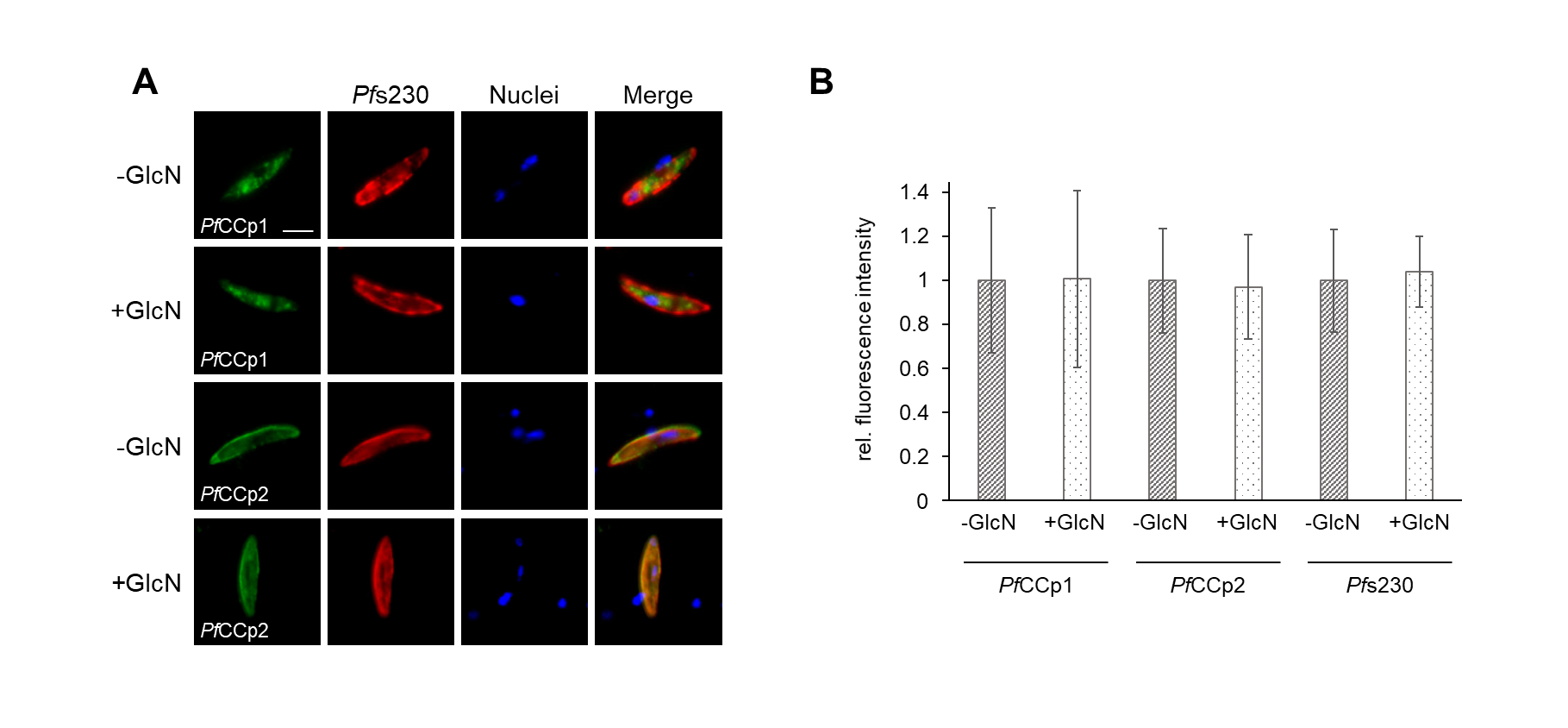


**Fig. S2** GlcN-treatment does not affect *Pf*CCp1 and *Pf*CCp2 abundance in WT NF54 gametocytes. **A.** Immunolabeling of *Pf*CCp1 and *Pf*CCp2 in gametocytes of WT NF54. WT NF54 gametocytes were cultivated either in the presence or absence of GlcN for 14 d. Untreated and GlcN-treated gametocytes were immunolabeled with polyclonal mouse anti-*Pf*CCp1 antiserum or polyclonal rabbit anti-*Pf*CCp2 antiserum (green) and counterstained with polyclonal rabbit or mouse anti-*Pf*s230 antisera (red). Nuclei were highlighted with Hoechst33342 nuclear stain (blue). Bar, 5 µm. The results are representative for three independent experiments. **B.** Immunolabeling has been performed as described in A. The fluorescence intensity of *Pf*CCp1, *Pf*CCp2 and *Pf*s230 signals was quantified in 20 gametocytes using ImageJ 1.51f (mean ± SD; untreated set to 1). The results are representative for two independent experiments.
